# Prediction of cognitive and motor outcome of preterm infants based on automatic quantitative descriptors from neonatal MR brain images

**DOI:** 10.1038/s41598-017-02307-w

**Published:** 2017-05-19

**Authors:** Pim Moeskops, Ivana Išgum, Kristin Keunen, Nathalie H. P. Claessens, Ingrid C. van Haastert, Floris Groenendaal, Linda S. de Vries, Max A. Viergever, Manon J. N. L. Benders

**Affiliations:** 1 Image Sciences Institute Department of Radiology University Medical Center Utrecht Heidelberglaan, 100 3584 CX Utrecht, The Netherlands; 2 Medical Image Analysis Group Department of Biomedical Engineering Eindhoven University of Technology, PO Box 513 5600 MB Eindhoven, The Netherlands; 3 Department of Neonatology Wilhelmina’s Children’s Hospital University Medical Center Utrecht Lundlaan, 6 3584 EA Utrecht, The Netherlands

## Abstract

This study investigates the predictive ability of automatic quantitative brain MRI descriptors for the identification of infants with low cognitive and/or motor outcome at 2–3 years chronological age. MR brain images of 173 patients were acquired at 30 weeks postmenstrual age (PMA) (n = 86) and 40 weeks PMA (n = 153) between 2008 and 2013. Eight tissue volumes and measures of cortical morphology were automatically computed. A support vector machine classifier was employed to identify infants who exhibit low cognitive and/or motor outcome (<85) at 2–3 years chronological age as assessed by the Bayley scales. Based on the images acquired at 30 weeks PMA, the automatic identification resulted in an area under the receiver operation characteristic curve (AUC) of 0.78 for low cognitive outcome, and an AUC of 0.80 for low motor outcome. Identification based on the change of the descriptors between 30 and 40 weeks PMA (n = 66) resulted in an AUC of 0.80 for low cognitive outcome and an AUC of 0.85 for low motor outcome. This study provides evidence of the feasibility of identification of preterm infants at risk of cognitive and motor impairments based on descriptors automatically computed from images acquired at 30 and 40 weeks PMA.

## Introduction

Brain MRI is an important tool to assess brain development and to predict neurodevelopmental outcome in preterm infants, as preterm birth is recognised to affect cognitive and motor development^1–8^. Many studies have shown relations between quantitative descriptors from brain MRI and cognitive and/or motor outcome of preterm born children or adolescents, i.e. with neuroimaging and neurodevelopmental outcome data acquired at the same age^9–11^. Additionally, several studies have shown relations between MRI descriptors at term-equivalent age and cognitive and/or motor outcome at a later age. These associations have been demonstrated using tissue volumes and morphometry from anatomical MRI^12–18^, and descriptors from diffusion MRI^17, 18^. Furthermore, Rathbone *et al*.^19^ showed associations between cognitive outcome and growth of the cortical surface area from images acquired between 24 and 44 weeks postmenstrual age (PMA).

However, to the best of our knowledge, no study has been performed that investigates the predictive ability of early quantitative brain MRI descriptors, such as volumes and measures of cortical morphology, for cognitive and motor outcome at 2–3 years chronological age. Early MRI is performed at around 30 weeks PMA—a few weeks after birth and as soon as clinical stability is reached—i.e. about ten weeks earlier than the term-equivalent age. Early identification of infants at risk of impairments might be especially valuable because it may fall within a window of opportunity for neuroprotective intervention. Furthermore, most studies investigated associations that are used to evaluate which descriptors are most prominently related to cognitive and/or motor outcome. However, in order to identify infants at risk of neurodevelopmental deficits, prediction models are required.

The purpose of this study is to investigate the predictive ability of automatic quantitative brain MRI descriptors acquired at 30 weeks PMA (the earliest postnatal stages in which neuroimaging is currently feasible in a cohort of preterm infants) and at 40 weeks PMA (the term-equivalent age), both separately and combined, for cognitive and/or motor outcome at 2–3 years chronological age.

## Materials and Methods

### Data

A cohort of 201 preterm infants born before 28 weeks gestational age (GA) was imaged on a Philips (Best, The Netherlands) Achieva 3T scanner between 2008 and 2013. MRI is a standard clinical care procedure at our institution performed around 30 and 40 weeks PMA in these infants owing to a high risk of brain injury and aberrant brain development. Given the fact that neuroimaging data were obtained as part of the clinical protocol, written informed consent for use of the clinically acquired data and approval of the experiments and methodology was waived by the institutional review board of the University Medical Center Utrecht, The Netherlands.

At 30 weeks PMA, infants were scanned in an MR compatible incubator (LMT Medical Systems, Lübeck, Germany) if considered clinically stable at the discretion of the attending neonatologist. Coronal T_2_-weighted images were acquired (n = 138) at an average PMA of 30.8 ± 0.8 weeks using a multislice turbo spin echo sequence (echo time: 120 ms, repetition time: 10085 ms, scan time: 6.23 min, field of view 104 × 130 × 100 mm^3^, no slice gap). The images were reconstructed to a matrix of 384 × 384 × 50 voxels with voxel sizes of 0.34 × 0.34 × 2.0 mm^3^.

At 40 weeks PMA the infants were scanned using a Philips SENSE head coil. Coronal T_2_-weighted images were acquired (n = 191) at an average PMA of 41.2 ± 0.7 weeks using a multislice turbo spin echo sequence (echo time: 150 ms, repetition time: 4847 ms, scan time: 5.05 min, field of view 180 × 180 × 132 mm^3^, no slice gap). The images were reconstructed to a matrix of 512 × 512 × 110 voxels with voxel sizes of 0.35 × 0.35 × 1.2 mm^3^.

Infants were always sedated using oral chloral hydrate 15 minutes prior to scanning at 40 weeks PMA (50–60 mg/kg) and at 30 weeks PMA at the discretion of the attending neonatologist or physician assistant (30 mg/kg). Heart rate, respiration rate and oxygen saturation levels were monitored throughout the procedure by a neonatologist or physician assistant who was always present during the MRI.

**Figure 1:**
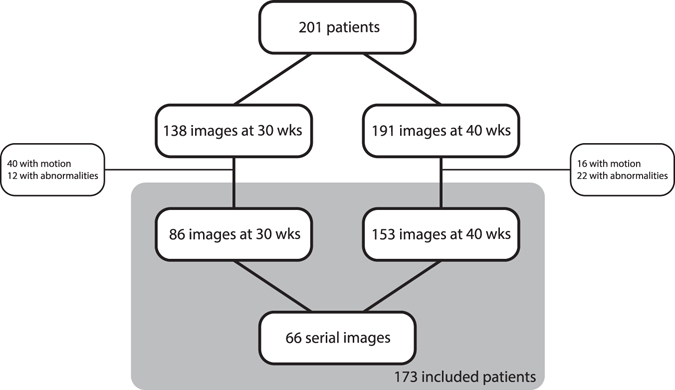
Exclusion of images because of motion artefacts or abnormalities that resulted in inaccurate automatic analysis.

Patients with brain abnormalities (including e.g. intraventricular haemorrhages, posthaemorrhagic ventricular dilation and cystic periventricular leukomalacia) and images with motion artefacts were excluded *only* if this resulted in inaccurate automatic analysis (Fig. 1). This resulted in inclusion of 86 MR images acquired at 30 weeks PMA and 153 MR images acquired at 40 weeks PMA from 173 different patients; 66 of the included patients had both MR images. Table 1 lists clinical data of the patients included and the patients excluded in the study.

**Table 1:** Patient characteristics and the number of patients with brain injury included and excluded in this study.

	Included patients	Excluded patients
Number of patients	173	28
Male/female	88/85	16/12
GA at birth (*μ* ± *σ*)	26.5 ± 1.0 weeks, range: 24–28 weeks	26.5 ± 0.8 weeks, range: 25–28 weeks
Birth weight (*μ* ± *σ*)	884 ± 169 g, range: 455–1330 g	963 ± 218 g, 515–1450 g
Prolonged mechanical ventilation (>7 days)	71 (41%)	20 (71%)
Intraventricular haemorrhage grade I–II	50 (29%)	4 (14%)
Intraventricular haemorrhage grade III	9 (5%)	3 (11%)
Intraventricular haemorrhage grade IV	2 (1%)	11 (39%)
Posthaemorrhagic ventricular dilation	3 (2%)	7 (25%)
Severe cerebellar haemorrhage	1 (1%)	4 (14%)
Cystic periventricular leukomalacia	1 (1%)	0 (0%)
Maternal education: Low	42 (24%)	6 (21%)
Maternal education: Middle	66 (38%)	13 (46%)
Maternal education: High	65 (38%)	9 (32%)

All patients included in this study were assessed using the Bayley scales of infant development, third edition (BSID-III)^20^ by a special educator at the outpatient clinic of the University Medical Center Utrecht at an average chronological age of 29 ± 3 months (range: 26–35 months). The scores were computed according to the Dutch reference values^21, 22^. Low cognitive outcome, i.e. the positive class, was defined as a cognitive composite score < 85, and low motor outcome was defined as a motor composite score < 85. Favourable outcome, i.e. the negative class, was defined as a cognitive and/or motor score ≥ 85. For cognitive outcome this division resulted in: 18 of the 86 patients with low outcome (21%) for the images acquired at 30 weeks PMA, 37 of the 153 patients with low outcome (24%) for the images acquired at 40 weeks PMA, and 13 of the 66 patients with low outcome (20%) for the serial images. For motor outcome this division resulted in: 15 of the 86 patients with low outcome (17%) for the images acquired at 30 weeks PMA, 38 of the 153 patients with low outcome (25%) for the images acquired at 40 weeks PMA, and 9 of the 66 patients with low outcome (14%) for the serial images.

### Methods

All 239 images (86 at 30 weeks PMA and 153 at 40 weeks PMA) were automatically segmented and quantitative descriptors were computed from the segmentations. Based on these descriptors a classifier was trained to identify patients with a low cognitive or motor outcome. The individual steps included in this process are outlined below.

#### Segmentations

All images were automatically segmented i﻿nto eight tissue types using a deep learning approach^23^. These tissue types included: (1) unmyelinated (uWM) and (2) myelinated white matter (mWM), (3) cortical grey matter (cGM), (4) ventricular (vCSF) and (5) extracerebral cerebrospinal fluid (eCSF), (6) cerebellum (CB), (7) basal ganglia and thalami (BGT), and (8) brain stem (BS). The segmentation method is based on a convolutional neural network that uses information from the coronal, axial and sagittal planes to label each voxel as one of the eight classes. The automatic method is trained using a set of ten manually segmented images acquired at 30 weeks PMA and using a set of five manually segmented images acquired at 40 weeks PMA. The detailed protocol of these manual segmentations has been described by Išgum *et al*.^24^.

**Figure 2:**
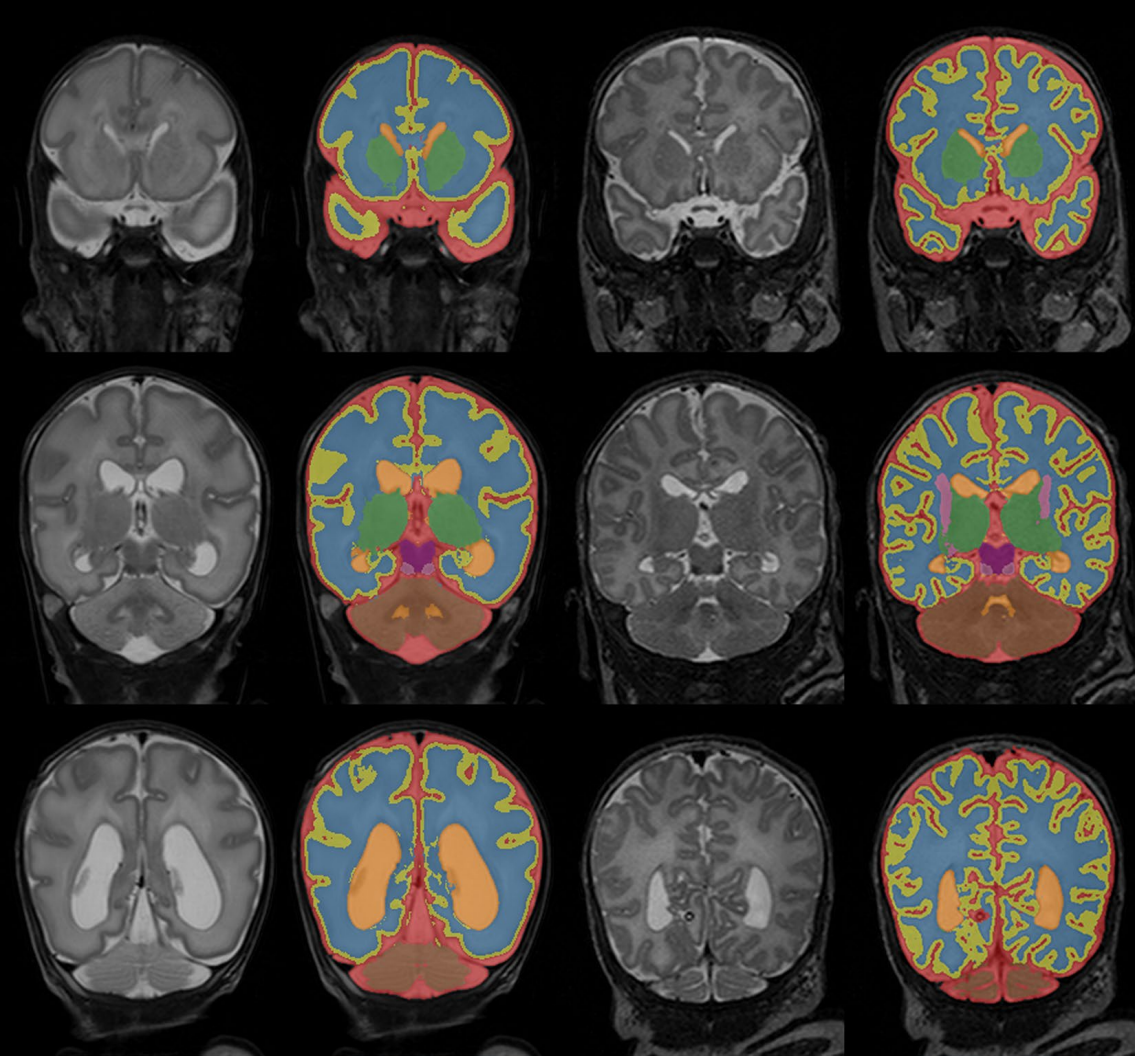
Automatic segmentation of the extracerebral cerebrospinal fluid (red), cortical grey matter (yellow), unmyelinated white matter (blue), ventricular cerebrospinal fluid (orange), basal ganglia and thalami (green), brain stem (purple), myelinated white matter (pink), and cerebellum (brown) in three slices of the images acquired at 30 (left) and 40 (right) weeks PMA for one patient. The images are scaled and cropped separately for better visualisation and do therefore not represent differences in size between 30 and 40 weeks PMA.

Figure 2 shows the automatic segmentation results for images acquired at both time points of a single infant.

#### Quantitative descriptors

Based on the automatic segmentations, a set of quantitative descriptors was computed for each image. First, relative volumes for each of the eight tissue classes were computed as the ratio between the tissue volume and total intracranial volume. Second, inner cortical surface area, gyrification index, global mean curvature, and median cortical thickness were computed. These descriptors were previously found to be related to PMA at imaging and brain injury in this population^25^. In addition, GA at birth was included in the analysis.

This resulted in a set of 14 descriptors: eight relative tissue volumes, intracranial volume, four measures of cortical morphology, and GA. Before classification all features were scaled between 0 and 1, estimated on the training set.

#### Classification

Support vector machine (SVM) classifiers^26^ with linear kernels were used to classify patients into two classes: those with an outcome below 85 and those with an outcome of 85 or higher. The classifiers fit a hyperplane in a multidimensional feature space to best discriminate between these two classes based on a training set. Therefore, the classification can be used to identify patients at risk of mild to severe cognitive/motor impairments. SVM classifiers are commonly used in prediction studies based on neuroimaging data, including the prediction of mild cognitive impairment, Alzheimer’s disease, depressive disorders, schizophrenia, autism spectrum disorder and attention-deficit hyperactivity disorder^27^.

Experiments were performed using (1) the descriptors from the images acquired at 30 weeks PMA, (2) the descriptors from images acquired at 40 weeks PMA, and (3) the differences between the descriptors from the serial images acquired at 30 and 40 weeks PMA, indicating the development during these ten weeks in terms of the descriptors, i.e. brain growth and cortical development. Seven different sets of descriptors were evaluated: first, all descriptors (volumes and measures of cortical morphology) were included, second, only volumes were included, and third, only measures of cortical morphology were included. Each of these sets of descriptors were evaluated with and without GA, in addition to the evaluation of GA as the only input feature. In addition to these seven predefined sets of descriptors, a full search of all possible combinations of descriptors was performed. For the 14 descriptors that were used, this resulted in the evaluation of 16383 combinations. Because of the limited number of samples, cross-validation experiments were performed for each set of images. The classifications were performed in 500 combinations, where in each experiment 5% of the samples were randomly selected as test set and the remaining samples were used to train the classifier. Selecting multiple random combinations allows estimating robust overall performance measures, less influenced by the random division of the data, which allows fair comparison and ranking between different sets of descriptors. Moreover, this cross-validation approach is chosen over a leave-one-subject-out cross-validation approach to provide a more reliable estimate of the true predictive ability of the method. Probabilistic results of the SVM classifications were obtained with Platt scaling^28^. The classification was implemented with scikit-learn 0.17.

The classification results were compared using receiver operation characteristic (ROC) curves, and the area under the curve (AUC).

## Results

**Figure 3:**
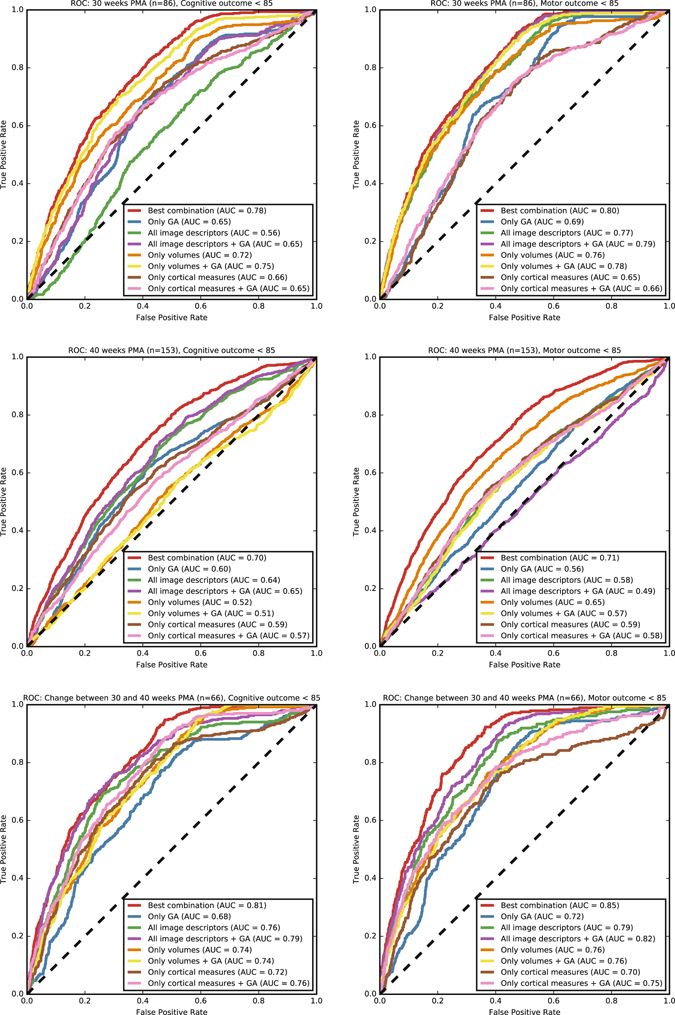
ROC curves for the prediction of patients with a cognitive (left) and motor (right) outcome below 85 as assessed by the BSID-III based on the images acquired at 30 weeks PMA (top), the images acquired at 40 weeks PMA (middle), and the change between the descriptors computed at 30 and 40 weeks PMA (bottom). The results are shown using: the best performing combinations from Tables 2, 3 and 4; only GA; all image descriptors; all image descriptors and GA; relative tissue volumes and intracranial volume; relative tissue volumes, intracranial volume, and GA; measures of cortical morphology; and measures of cortical morphology and GA.

Cross-validation experiments were performed using different subsets of descriptors to evaluate which were most predictive. First, all descriptors computed from the images (tissue volumes and measures of cortical morphology) were included; second, only tissue volumes and total intracranial volume were included; and third, only measures of cortical morphology were included. For each of these set of features, the predictive ability was evaluated with and without including GA at birth. Figure 3 shows the results for the images acquired at 30 weeks PMA (upper panels), the images acquired at 40 weeks PMA (middle panels), and the change of the descriptors between 30 and 40 weeks PMA (lower panels). Tables 2, 3 and 4 list the 10 best performing combinations of descriptors for each of the three inputs.

**Table 2:** AUCs for the 10 best performing combinations of descriptors for cognitive (top) and motor (bottom) outcome based on the images acquired at 30 weeks PMA.

**30 weeks PMA (n = 86), Cognitive outcome < 85**														
AUC	GA	CB	mWM	BGT	vCSF	uWM	BS	cGM	eCSF	ISA	MC	GI	MT	BV
0.775	×	×		×			×	×	×	×	×	×		×
0.774	×	×		×		×	×	×	×	×		×		×
0.774	×		×	×	×	×	×	×	×			×	×	
0.773	×	×	×	×		×	×	×	×		×	×	×	
0.773	×	×	×	×	×	×	×	×	×			×	×	
0.773	×	×		×	×	×	×	×		×	×		×	×
0.773	×	×	×			×	×	×	×		×	×	×	
0.772	×	×		×		×		×	×	×	×	×		×
0.772	×	×	×	×			×	×	×			×	×	
0.771	×	×			×	×		×	×	×	×	×		×
**Count**	10	9	5	8	4	8	8	10	9	5	6	9	6	5
**30 weeks PMA (n = 86), Motor outcome < 85**														
**AUC**	**GA**	**CB**	**mWM**	**BGT**	**vCSF**	**uWM**	**BS**	**cGM**	**eCSF**	**ISA**	**MC**	**GI**	**MT**	**BV**
0.797	×			×	×		×	×		×	×	×	×	×
0.797	×	×			×		×	×		×	×	×	×	×
0.797	×			×	×			×		×	×	×	×	×
0.796	×				×		×	×		×	×	×	×	×
0.795	×			×	×	×		×		×		×		×
0.795	×	×		×	×		×	×	×	×			×	×
0.773	×		×	×	×	×	×	×		×	×			×
0.772	×			×	×	×	×	×		×	×	×	×	×
0.772	×			×	×		×		×	×		×		×
0.771	×				×	×	×	×	×	×	×		×	×
**Count**	10	2	1	7	10	4	8	9	3	10	7	7	7	10

GA: gestational age, CB: cerebellum volume, mWM: myelinated white matter volume, BGT: basal ganglia and thalami volume, vCSF: ventricular cerebrospinal fluid volume, uWM: unmyelinated white matter volume, BS: brain stem volume, cGM: cortical grey matter volume, eCSF: extracerebral cerebrospinal fluid volume, ISA: inner cortical surface area, MC: global mean curvature, GI: gyrification index, MT: median cortical thickness, BV: brain volume. The bottom row shows in how many of the combinations each descriptor is present.

**Table 3:** AUCs for the 10 best performing combinations of descriptors for cognitive (top) and motor (bottom) outcome based on the images acquired at 40 weeks PMA.

**40 weeks PMA (n = 153), Cognitive outcome < 85**														
AUC	GA	CB	mWM	BGT	vCSF	uWM	BS	cGM	eCSF	ISA	MC	GI	MT	BV
0.702	×			×	×	×	×	×	×	×	×	×	×	×
0.689	×	×			×	×		×	×	×	×	×	×	×
0.686	×	×			×	×	×	×	×	×	×	×	×	×
0.686	×	×	×	×	×	×	×		×		×	×	×	
0.686				×	×	×	×	×	×	×	×	×	×	×
0.682	×	×		×	×	×	×	×		×	×	×	×	×
0.681	×			×	×	×		×	×	×	×	×	×	×
0.680	×	×		×	×	×	×		×	×	×	×	×	×
0.679	×		×	×	×	×	×	×	×	×	×	×		
0.677	×	×		×	×		×	×	×	×	×	×	×	×
**Count**	9	6	2	8	10	9	8	8	9	9	10	10	9	8
**40 weeks PMA (n = 153), Motor outcome < 85**														
**AUC**	**GA**	**CB**	**mWM**	**BGT**	**vCSF**	**uWM**	**BS**	**cGM**	**eCSF**	**ISA**	**MC**	**GI**	**MT**	**BV**
0.714		×	×		×	×	×		×	×	×	×	×	
0.714	×	×	×	×		×	×	×		×		×	×	×
0.713		×	×		×	×	×		×		×	×	×	×
0.712		×	×		×	×	×	×		×		×	×	×
0.712		×	×		×	×	×		×	×	×	×	×	×
0.711	×	×	×				×	×	×	×		×	×	×
0.711		×	×		×	×	×		×	×	×		×	×
0.710		×	×		×		×	×		×	×	×	×	×
0.709	×		×			×	×	×	×	×	×	×	×	×
0.709	×	×	×	×		×	×	×		×		×		×
**Count**	4	9	10	2	6	8	10	6	6	9	6	9	9	9

GA: gestational age, CB: cerebellum volume, mWM: myelinated white matter volume, BGT: basal ganglia and thalami volume, vCSF: ventricular cerebrospinal fluid volume, uWM: unmyelinated white matter volume, BS: brain stem volume, cGM: cortical grey matter volume, eCSF: extracerebral cerebrospinal fluid volume, ISA: inner cortical surface area, MC: global mean curvature, GI: gyrification index, MT: median cortical thickness, BV: brain volume. The bottom row shows in how many of the combinations each descriptor is present.

**Table 4:** AUCs for the 10 best performing combinations of descriptors for cognitive (top) and motor (bottom) outcome based on the change between 30 and 40 weeks PMA.

**Change between 30 and 40 weeks PMA (n = 66), Cognitive outcome < 85**														
AUC	GA	CB	mWM	BGT	vCSF	uWM	BS	cGM	eCSF	ISA	MC	GI	MT	BV
0.814		×	×	×	×	×	×		×	×	×	×	×	×
0.814	×	×		×	×	×	×		×	×		×	×	×
0.814		×		×	×	×	×			×		×		×
0.813	×	×	×	×	×	×	×		×	×	×	×		×
0.813		×		×	×	×	×			×	×	×		
0.813	×	×		×	×	×	×		×	×	×	×	×	×
0.812	×	×	×	×	×	×	×		×	×		×	×	×
0.812	×		×	×	×	×	×		×	×	×	×	×	×
0.812	×	×			×	×	×			×	×			×
0.811		×		×	×	×	×			×		×	×	
**Count**	6	9	4	9	10	10	10	0	6	10	6	9	6	8
**Change between 30 and 40 weeks PMA (n = 66), Motor outcome < 85**														
**AUC**	**GA**	**CB**	**mWM**	**BGT**	**vCSF**	**uWM**	**BS**	**cGM**	**eCSF**	**ISA**	**MC**	**GI**	**MT**	**BV**
0.847	×		×	×	×			×		×	×	×	×	×
0.847	×		×	×	×	×		×	×	×		×	×	×
0.846	×		×	×	×	×		×		×	×	×	×	×
0.846			×	×	×		×	×		×		×	×	×
0.846	×		×	×	×			×			×	×	×	×
0.845	×			×	×			×	×		×	×	×	×
0.844	×		×	×	×	×		×		×		×	×	×
0.844	×		×	×	×			×	×	×		×	×	×
0.843			×	×	×		×	×		×		×		×
0.843	×			×	×			×	×	×	×	×	×	
**Count**	8	0	8	10	10	3	2	10	4	8	5	10	9	9

GA: gestational age, CB: cerebellum volume, mWM: myelinated white matter volume, BGT: basal ganglia and thalami volume, vCSF: ventricular cerebrospinal fluid volume, uWM: unmyelinated white matter volume, BS: brain stem volume, cGM: cortical grey matter volume, eCSF: extracerebral cerebrospinal fluid volume, ISA: inner cortical surface area, MC: global mean curvature, GI: gyrification index, MT: median cortical thickness, BV: brain volume. The bottom row shows in how many of the combinations each descriptor is present.

For the identification of patients with low cognitive outcome based on the images acquired at 30 weeks PMA, the highest AUC (0.78) was obtained using the combination of GA, CB volume, BGT volume, BS volume, cGM volume, eCSF volume, inner cortical surface area, global mean curvature, gyrification index, and brain volume (Table 2 and Fig. 3, top left panel). Using the predefined sets of descriptors, the highest AUC (0.75) was obtained using a combination of the volumes and GA. Using the whole set of descriptors (volumes and measures of cortical morphology) decreased the AUC (0.56 without GA and 0.65 with GA); using the measures of cortical morphology alone also resulted in a lower AUC (0.66 without GA and 0.65 with GA). The AUC was lower for the descriptors computed from the images acquired at 40 weeks PMA (Table 3 and Fig. 3, middle left panel); the highest AUC (0.70) was obtained using the combination of GA, BGT volume, vCSF volume, uWM volume, BS volume, cGM volume, eCSF volume, inner cortical surface area, global mean curvature, gyrification index, median cortical thickness, and brain volume. Using the predefined sets of descriptors, the highest AUC (0.65) was obtained using all descriptors combined. When the change of the descriptors between 30 and 40 weeks (n = 66) was used, higher AUCs were obtained (Table 4 and Fig. 3, bottom left panel); the highest AUC (0.81) was obtained using the combination of CB volume, mWM volume, BGT volume, vCSF volume, uWM volume, BS volume, eCSF volume, inner cortical surface area, global mean curvature, gyrification index, median cortical thickness, and brain volume. Using the predefined sets of descriptors, the highest AUC (0.79) was obtained using all descriptors (including GA); the AUCs using only measures of cortical morphology (0.76) and using only volumes (0.74) were lower.

For the identification of patients with low motor outcome based on the images acquired at 30 weeks PMA, the highest AUC (0.80) was obtained using the combination of GA, BGT volume, vCSF volume, BS volume, cGM volume, inner cortical surface area, global mean curvature, gyrification index, median cortical thickness, and brain volume (Table 2 and Fig. 3, top right panel). Using the predefined sets of descriptors, the highest AUC (0.79) was obtained when using the combination of all descriptors (including GA). Using only volumes resulted in similar AUCs (0.76 without GA and 0.78 with GA). However, using only measures of cortical morphology resulted in lower AUC (0.65 without GA and 0.66 with GA). Similar to the results for cognitive outcome, the AUC was lower for the descriptors computed from the images acquired at 40 weeks PMA; the highest AUC (0.71) was obtained using the combination of CB volume, mWM volume, vCSF volume, uWM volume, BS volume, eCSF volume, inner cortical surface area, global mean curvature, gyrification index, and median thickness (Table 3 and Fig. 3, middle right panel). Using the predefined sets of descriptors, the highest AUC (0.65) was obtained using volumes only, including the GA decreased the AUC (0.57). When the change between the descriptors of the serial images (n = 66) was used, the highest AUC (0.85) was obtained using the combination of GA, mWM volume, BGT volume, vCSF volume, cGM volume, inner cortical surface area, global mean curvature, gyrification index, median cortical thickness, and brain volume. Using the predefined sets of descriptors, the highest AUC (0.82) was obtained using the combination of all descriptors (including GA) (Fig. 3, bottom right panel); the AUCs using only volumes (0.76) and using only measures of cortical morphology (0.75) were lower.

## Discussion

This paper presented a neonatal brain MRI prediction approach that is able to identify preterm infants at risk of cognitive and motor impairment at 2–3 years chronological age based exclusively on quantitative descriptors automatically computed from MR images acquired at 30 and 40 weeks PMA, i.e. without including clinical information.

The results suggest that the prediction could be based exclusively on the images acquired at 30 weeks PMA, well before term-equivalent age, with an AUC of 0.78 for cognitive outcome and an AUC of 0.80 for motor outcome (Fig. 3, first row). The predictive ability of the images acquired at 40 weeks PMA alone appears lower, with an AUC of 0.70 for cognitive outcome and an AUC of 0.71 for motor outcome (Fig. 3, second row). The observation that neurodevelopmental outcome could best be predicted from neonatal MRI at 30 weeks PMA is especially valuable because identification of infants at risk at around 30 weeks PMA may fall within or even before a window of opportunity for neuroprotective intervention.

Using the change of the descriptors between 30 and 40 weeks PMA resulted in a higher performance, with an AUC of 0.81 for cognitive outcome and an AUC of 0.85 for motor outcome (Fig. 3, third row). The better predictive ability based on the change in descriptors between 30 and 40 weeks PMA could be explained by the rapid development of especially the cortex during these 10 weeks, which is therefore very vulnerable in extra-uterine conditions. This development is commonly hypothesised to be associated with cognitive functions^19, 29, 30^, and could therefore be a better indicator of the neurodevelopmental state than descriptors acquired at single time points.

Overall, the most common descriptor over Tables 2, 3 and 4 is gyrification index, which is present in 54 of the 60 best performing sets of descriptors. Other common descriptors in these best performing sets of descriptors are: inner cortical surface area (51/60), vCSF volume (50/60), and brain volume (49/60). Each of these descriptors indicate a specific part of brain development: gyrification index and inner cortical surface area could e.g. indicate impaired development of cortical folding, vCSF volume could e.g. indicate enlarged ventricles because of brain injury, and total brain volume could e.g. indicate impaired overall brain growth. Another important observation is that the number of descriptors in each of the best performing sets is between 8 and 12, indicating that the predictive ability is likely in combinations of descriptors instead of single descriptors.

Infants with a low GA at birth are recognised to have a higher risk for impairments^3, 5^. GA at birth also appeared relevant for the identification of patients with low outcome in our experiments: inclusion in the set of descriptors resulted in most of the cases in a (slightly) higher predictive ability than when only using the MRI descriptors. GA is furthermore present in each of the 10 best performing sets of descriptors based on the images acquired at 30 weeks PMA, for both cognitive and motor outcome (Table 2), and in total present in 47 of the 60 sets in Tables 2, 3 and 4. Nevertheless, even without including GA, prediction was possible based on the MRI descriptors only.

The results underscore the importance of image analysis and machine learning methods to improve neurodevelopmental outcome predictions. In the literature, severe impairments following preterm birth, especially in the motor domains, have been consistently associated with early imaging biomarkers, yet making accurate predictions with respect to (mild) cognitive deficits remains notoriously difficult^13, 31–33^. These deficits are clinically relevant as they comprise the majority of neurodevelopmental impairments that preterm born children are confronted with and have significant impact on the wellbeing of the child and its family as well as on society. Fully automatic quantification and prediction using machine learning as employed in our study may comprise promising tools towards improving diagnosis and prognostication of infants at risk of impairments. Consequently, early diagnosis would allow for early intervention strategies such as rehabilitation therapy or educational support.

There is agreement in the literature that the BSID-III tends to overestimate developmental status in preterm infants^34^. Using a corrected age with the cut-off value of 85 (−1 standard deviation) may underestimate the number of infants with an impaired outcome, especially in a rather healthy population with a good outcome like ours. We therefore decided to use the chronological age rather than the corrected age. This resulted in 24% of the 173 infants with low cognitive outcome and 25% of the 173 infants with low motor outcome, which corresponds to the percentage of children classified with low outcome at school-age in other studies^35^. Future work could focus on regression instead of classification, i.e. prediction of the actual outcome score instead identification of infants with low outcome, which was not reliable based on the number of patients included in the present study.

This study has limitations. First, the number of included patients was relatively small for a prediction study (86 MR images acquired at 30 weeks PMA, 153 MR images acquired at 40 weeks PMA, and 66 MR images acquired at both time points). But considering that the data were derived from a complex patient population and included serial imaging as well as outcome data, the data set was relatively large. In order to account for the small sample size, cross-validation experiments in multiple combinations were performed to provide an estimate of the predictive ability. However, validation studies in larger (multi-centre) cohorts are necessary to further validate the results and evaluate whether the results generalise well to different patient populations. Nevertheless, the results indicate that valuable information for the prediction of cognitive and motor outcome later in life can be fully automatically extracted based on the images only. Second, given the fact that neuroimaging data were acquired as part of routine care and patients with severe abnormalities that resulted in unreliable image quantification were excluded, a selection bias occurred towards the healthier and more mature infants. Third, the average follow-up time (2–3 years chronological age) is relatively short; outcome data at school age are likely to provide a better estimation of especially cognitive development and would be relevant to verify whether these findings remain present in childhood as well.

This study provides evidence of the feasibility of identification of preterm infants at risk of both cognitive and motor impairments based on the change of quantitative descriptors automatically computed from images acquired at 30 and 40 weeks PMA. We furthermore show that the identification could even be based exclusively on an image acquired at 30 weeks PMA. Future studies should include larger sample sizes (across multiple study sites) and follow-up data through school age to corroborate these findings.

## References

[1] Saigal S, Doyle LW (2008). An overview of mortality and sequelae of preterm birth from infancy to adulthood. Lancet.

[2] Larroque B (2011). Special care and school difficulties in 8-year-old very preterm children: The Epipage Cohort study. PLoS One.

[3] Moore T (2012). Neurological and developmental outcome in extremely preterm children born in England in 1995 and 2006: the EPICure studies. BMJ Br. Med. J..

[4] Keunen K (2012). Brain tissue volumes in preterm infants: prematurity, perinatal risk factors and neurodevelopmental outcome: A systematic review. J. Matern. Neonatal Med..

[5] Serenius F (2013). Neurodevelopmental Outcome in Extremely Preterm Infants at 2.5 Years After Active Perinatal Care in Sweden. JAMA.

[6] Marret S (2013). Brain Injury in Very Preterm Children and Neurosensory and Cognitive Disabilities during Childhood: The EPIPAGE Cohort Study. PLoS One.

[7] Hintz SR (2015). Neuroimaging and neurodevelopmental outcome in extremely preterm infants. Pediatrics.

[8] Bouyssi-Kobar M (2016). Third Trimester Brain Growth in Preterm Infants Compared With In Utero Healthy Fetuses. Pediatrics.

[9] de Kieviet JF, Zoetebier L, van Elburg RM, Vermeulen RJ, Oosterlaan J (2012). Brain development of very preterm and very low-birthweight children in childhood and adolescence: a meta-analysis. Dev. Med. Child Neurol..

[10] Cheong JLY (2013). Contribution of Brain Size to IQ and Educational Underperformance in Extremely Preterm Adolescents. PLoS One.

[11] Allin M (2001). Cognitive and motor function and the size of the cerebellum in adolescents born very pre-term. Brain.

[12] Van Kooij BJM (2012). Cerebellar volume and proton magnetic resonance spectroscopy at term, and neurodevelopment at 2 years of age in preterm infants. Dev. Med. Child Neurol..

[13] Keunen K (2016). Brain Volumes at Term-Equivalent Age in Preterm Infants: Imaging Biomarkers for Neurodevelopmental Outcome through Early School Age. J. Pediatr..

[14] Hansen-Pupp I (2013). Circulatory insulin-like growth factor-I and brain volumes in relation to neurodevelopmental outcome in very preterm infants. Pediatr. Res..

[15] Lind A (2011). Associations between regional brain volumes at term-equivalent age and development at 2 years of age in preterm children. Pediatr. Radiol..

[16] Peterson BS (2003). Regional brain volumes and their later neurodevelopmental correlates in term and preterm infants. Pediatrics.

[17] Skiöld B (2014). Sex differences in outcome and associations with neonatal brain morphology in extremely preterm children. J. Pediatr..

[18] Ullman H (2015). Neonatal MRI is associated with future cognition and academic achievement in preterm children. Brain.

[19] Rathbone R (2011). Perinatal cortical growth and childhood neurocognitive abilities. Neurology.

[20] Bayley, N. *Bayley Scales of Infant and Toddler Development*, *Third Edition*. (Harcourt Assessment, Inc., 2006).

[21] van Baar, A. L., Steenis, L. J. P., Verhoeven, M. & Hessen, D. J. *Bayley-III-NL, Supplement Handmatige Scoring*. (Pearson Assessment and Information B.V., 2014).

[22] Steenis LJP, Verhoeven M, Hessen DJ, van Baar AL (2015). Performance of Dutch children on the Bayley III: a comparison study of US and Dutch norms. PLoS One.

[23] Moeskops P (2016). Automatic segmentation of MR brain images with a convolutional neural network. IEEE Trans. Med. Imaging.

[24] Išgum I (2015). Evaluation of automatic neonatal brain segmentation algorithms: The NeoBrainS12 challenge. Med. Image Anal..

[25] Moeskops P (2015). Development of Cortical Morphology Evaluated with Longitudinal MR Brain Images of Preterm Infants. PLoS One.

[26] Cortes C, Vapnik V (1995). Support-vector networks. Mach. Learn..

[27] Arbabshirani MR, Plis S, Sui J, Calhoun VD (2017). Single subject prediction of brain disorders in neuroimaging: Promises and pitfalls. Neuroimage.

[28] Platt J (1999). Probabilistic outputs for support vector machines and comparisons to regularized likelihood methods. Adv. large margin Classif..

[29] Ajayi-Obe M, Saeed N, Cowan F, Rutherford M, Edwards D (2000). Reduced development of cerebral cortex in extremely preterm infants. Lancet.

[30] Dubois J (2008). Primary cortical folding in the human newborn: an early marker of later functional development. Brain.

[31] Woodward LJ, Anderson PJ, Austin NC, Howard K, Inder TE (2006). Neonatal MRI to predict neurodevelopmental outcomes in preterm infants. N. Engl. J. Med..

[32] Thompson DK (2012). Corpus callosum alterations in very preterm infants: Perinatal correlates and 2 year neurodevelopmental outcomes. Neuroimage.

[33] Van Kooij B (2012). Neonatal Tract-Based Spatial Statistics Findings and Outcome in Preterm Infants. Am. J. Neuroradiol..

[34] Spencer-Smith MM, Spittle aJ, Lee KJ, Doyle LW, Anderson PJ (2015). Bayley-III Cognitive and Language Scales in Preterm Children. Pediatrics.

[35] Larroque B (2008). Neurodevelopmental disabilities and special care of 5-year-old children born before 33 weeks of gestation (the EPIPAGE study): a longitudinal cohort study. Lancet.

